# Impact of 2.5 mg versus 5 mg letrozole co-treatment in an antagonist protocol for IVF: a retrospective study

**DOI:** 10.3389/fendo.2023.1289595

**Published:** 2023-11-10

**Authors:** Jing Lin, Fenglu Wu, Kai Zhang, Yanwen Zhu, Bian Wang, Qianqian Zhu, Jiaying Lin

**Affiliations:** ^1^ Center for Reproductive Medicine, Xinhua Hospital, Shanghai Jiao Tong University School of Medicine, Shanghai, China; ^2^ Department of Assisted Reproduction, Shanghai Ninth People’s Hospital, Shanghai Jiao Tong University School of Medicine, Shanghai, China; ^3^ China National Cancer Center/National Clinical Research Center for Cancer/Hebei Cancer Hospital, Chinese Academy of Medical Sciences, Langfang, China

**Keywords:** letrozole, antagonist protocol, oestradiol, live birth, *in vitro* fertilization, assisted reproductive technology

## Abstract

**Objective:**

The present study aimed to compare the effectiveness of two different doses of letrozole (2.5 mg and 5 mg daily) in an antagonist protocol for infertile women with normal ovarian reserve.

**Methods:**

This retrospective cohort study included infertile women who underwent *in vitro* fertilization treatment with letrozole co-treatment at doses of 2.5 mg and 5 mg from 2007 – 2021 at Shanghai Ninth People’s Hospital (Shanghai, China). The control group comprised infertile women who received gonadotropin-releasing hormone antagonist alone. The primary outcome was the cumulative live birth rate, while secondary outcomes included follicular phase endocrine parameters, ovarian stimulation outcomes, pregnancy outcomes, and the incidences of maternal and neonatal complications. Baseline and follow-up data were compared between the groups using ANOVA for normally distributed variables, the Kruskal-Wallis test for non-normally distributed variables, and the Chi-square test for categorical variables.

**Results:**

A total of 422 participants were enrolled in the study, with 211 women in the antagonist group, 109 women in the 2.5 mg letrozole co-treatment group, and 102 women in the 5 mg letrozole co-treatment group. Letrozole co-treatment significantly suppressed oestradiol and follicle-stimulating hormone concentrations from stimulation day 5 and onwards, while increasing luteinizing hormone levels on stimulation day 5 and trigger day. The effect was more pronounced with a 5 mg dose of letrozole compared to a 2.5 mg dose (P < 0.05). Administration of 5 mg letrozole reduced the gonadotropin dose (P < 0.05) without negatively affecting the number of oocytes retrieved and subsequent embryo parameters (P > 0.05). The analysis of cumulative live birth rates showed rates of 29.4% in the letrozole 5 mg group, 27.5% in the letrozole 2.5 mg group, and 33.6% in the control group, with no statistically significant difference (P > 0.05). There were no reported pregnancy complications in the two letrozole groups. Additionally, there were no significant differences among the three groups in terms of gestational age and birth weight for both singleton and twin births.

**Conclusion:**

This study indicates that the administration of letrozole in an antagonist protocol, at both 2.5 mg and 5 mg dosages, results in comparable clinical outcomes.

## Introduction

Letrozole, a third-generation aromatase inhibitor, effectively reduces both intraovarian and serum estrogen levels by inhibiting the conversion of androgens to estrogens in ovarian granulosa cells (1). This mechanism allows for the maintenance of follicular phase oestradiol levels closer to physiological levels. The addition of letrozole to gonadotropin stimulation protocols has been widely accepted as a treatment option for oocyte retrieval in women with estrogen-sensitive tumors, such as breast cancer (2, 3). For women with poor ovarian response, the combination of letrozole with a gonadotropin-releasing hormone (GnRH) antagonist protocol for *in vitro* fertilization (IVF) or intracytoplasmic sperm injection (ICSI) has been proposed to enhance follicular response and improve oocyte quality (4). Furthermore, in normal women undergoing IVF, lowering serum and follicular estrogen levels could potentially reduce the risk of ovarian hyperstimulation syndrome (OHSS) (5).

Recent research has highlighted the potential benefits of letrozole co-treatment extending into the luteal phase, alleviating the detrimental effects of cumulative oestradiol concentrations on both oocyte quality and endometrial receptivity (6, 7). This approach enables embryo transfer (ET) in a more natural hormonal and uterine environment. Previous studies have explored various doses of letrozole administered during IVF, ranging from 2.5 mg to 20 mg (8). At present, the most commonly used dosages in clinical practice are 2.5 mg and 5 mg. However, no research has investigated the comparative efficacy of 2.5 mg versus 5 mg letrozole during ovarian stimulation for IVF.

Therefore, the present study aims to compare the efficacy of different doses of letrozole (2.5 mg and 5 mg daily) in an antagonist protocol for infertile women with normal ovarian reserve.

## Materials and methods

The study was a retrospective cohort study approved by the Institutional Review Board of Shanghai Ninth People’s Hospital of Shanghai Jiao Tong University School of Medicine (Shanghai, China). Informed consent was waived as the data were deidentified and the analyses were retrospective in nature. This report was written in accordance with the Strengthening the Reporting of Observational Studies in Epidemiology (STROBE) reporting guidelines.

### Study participants

**Figure 1:**
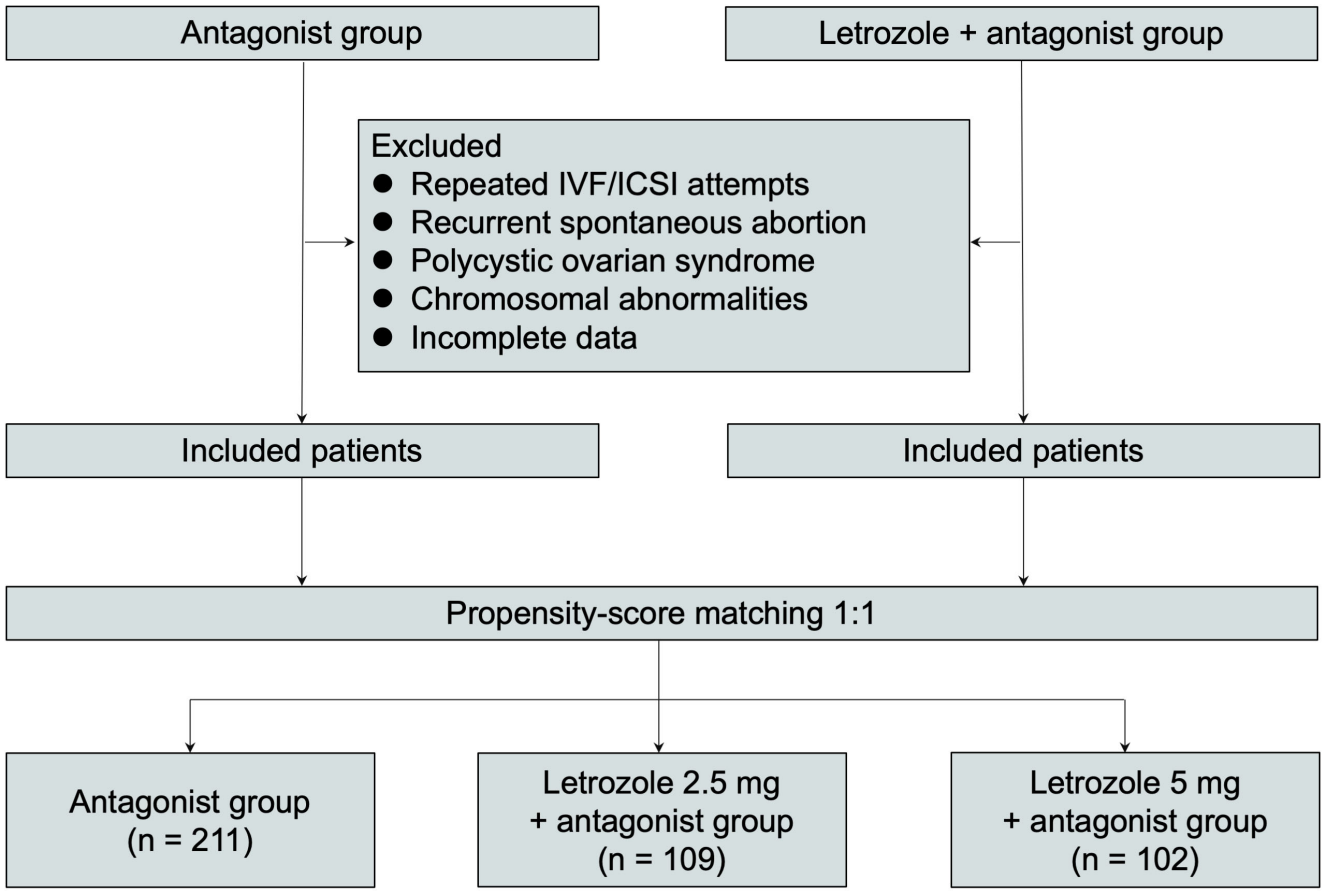
Study participant selection flowchart. *ICSI* intracytoplasmic sperm injection, *IVF* in vitro fertilization.

The study participants consisted of women undergoing IVF/ICSI treatment with co-administration of letrozole at doses of 2.5 mg or 5 mg in an antagonist protocol at Shanghai Ninth People’s Hospital between 2007 and 2021. The control group comprised infertile women who received GnRH antagonist alone during the same period. The control group was age-matched to the letrozole groups at a 1:1 ratio using a propensity-score matching approach. Inclusion criteria for women with expected normal ovarian reserve were as follows: age between 18 and 40 years, regular menstrual cycle between 21 and 35 days, antral follicle count (AFC) between 7 and 15, or follicle-stimulating hormone (FSH) less than 15 IU/l in the early follicular phase. Women with a history of repeated IVF/ICSI attempts, recurrent spontaneous abortion, polycystic ovarian syndrome according to the Rotterdam criteria, chromosomal abnormalities, or incomplete data were excluded. The final study population included 211 women in the GnRH antagonist group, 109 women in the 2.5 mg letrozole co-treatment group, and 102 women in the 5 mg letrozole co-treatment group (**Figure 1**).

### IVF/ICSI-ET procedures

All women underwent ovarian stimulation using an antagonist protocol. Gonadotropin administration began on cycle day 2-3 at a dose of 75-300 IU/day and continued until the human chorionic gonadotropin (hCG) trigger day. The dose was adjusted based on follicle growth and serum hormone levels. A daily injection of GnRH antagonist was initiated when a dominant follicle reached a mean diameter of 13-14 mm or when blood luteinizing hormone (LH) levels showed a notable upward trend. The antagonist was continued until the hCG trigger day. In the letrozole co-treatment group, patients received oral letrozole at a dosage of 2.5 mg/day or 5 mg/day for 5 consecutive days starting from cycle day 2-5.

Final oocyte maturation was triggered by the administration of triptorelin 0.1 mg and hCG 2000 IU when two leading follicles reached a diameter of 18 mm or more. Oocyte retrieval was performed 34-36 hours after hCG injection. Fertilization was achieved through IVF, ICSI, or IVF+ICSI, depending on sperm quality. Embryo quality was assessed according to the Istanbul Consensus. Fresh ET was preformed based on clinical practice, and any surplus embryos were cryopreserved for subsequent frozen-thawed embryo transfer (FET). Luteal support was provided after ET.

### Data collection and outcome measures

All patient data were extracted from electronic medical records. Demographic data included maternal age, pregestational body mass index (BMI), ethnicity, residency, gravidity, parity, infertility cause, infertility duration, and AFC. Serum concentrations of oestradiol, progesterone, FSH, and LH were collected at different time points during the stimulation period, including stimulation day 1 (SD1), 5-6 days after stimulation (SD5), 2-3 days before ovulation triggering (2dbTrigger), and on the day of ovulation trigger (Trigger). Ovarian stimulation outcomes were analyzed in terms of total gonadotropin consumption, duration of stimulation, number of oocytes retrieved, and high-quality embryos. Clinical pregnancy was defined as the presence of at least one intrauterine gestational sac identified on ultrasonography 35 days after ET. Live birth was defined as the delivery of at least one live-born infant regardless of gestational duration. The incidences of pregnancy complications, including hypertensive disorders in pregnancy, gestational diabetes, intrahepatic cholestasis of pregnancy, placental previa, placental abruption, and preterm premature rupture of the membranes, were evaluated for all live births resulting from ET cycles. Neonatal information, including gestational age, birth weight, and sex ratio, was collected.

The primary outcome of the study was the cumulative live birth rate, calculated with the fresh ET and all subsequent FETs resulting from the initial stimulation. The secondary outcomes included: (1) follicular phase endocrine parameters, (2) ovarian stimulation outcomes, (3) pregnancy rate and live birth rate per ET, (4) pregnancy complications, and (5) neonatal outcomes. Adverse events were recorded during clinic visits until a negative serum hCG test or through a telephone follow-up until fetal birth.

### Statistical analyses

The normality distribution of continuous variables was analyzed with the Shapiro-Wilk test. Normally distributed variables were presented as means (standard deviations), non-normally distributed variables as medians (interquartile ranges), and categorical variables as numbers (percentages). Baseline and follow-up data were compared between groups using ANOVA for normally distributed variables, the Kruskal-Wallis test for non-normally distributed variables, and the Chi-square test for categorical variables. A two-sided P-value of less than 0.05 was considered statistically significant.

Differences in repeated endocrine measurements at different time points were compared using a two-way mixed ANOVA on log-transformed concentrations, with a Bonferroni correction applied for multiple testing. To assess the pooled concentration of each endocrine parameter during the follicular phase, the area under the curve (AUC) was calculated using the hormone levels measured on multiple days (SD1, SD5, Trigger). The AUC was estimated using the trapezoid method, which involved summing the areas of trapezoids formed by consecutive hormone level measurements. Differences among the groups were evaluated using ANOVA on log-transformed AUC values, with a Bonferroni correction for multiple comparisons.

All statistical analyses were performed with R v 4.2.2. Data analyses were conducted between June and July 2023.

## Results

A total of 422 participants were enrolled in the study, comprising 211 women in the GnRH antagonist group, 109 women in the 2.5 mg letrozole co-treatment group, and 102 women in the 5 mg letrozole co-treatment group (**Figure 1**). The baseline characteristics of the three groups, including maternal age at oocyte retrieval, pregestational BMI, ethnicity, residency, gravidity, parity, infertility cause, and infertility duration, were found to be comparable (P > 0.05), as summarized in **Table 1**. Women in the letrozole 5 mg co-treatment group tended to have lower AFC compared to the control group (7 [3-10] versus 8 [5-11]; P < 0.05).

**Table 1:** Study participant characteristics.

	GnRH antagonist (N = 211)	Letrozole 2.5 mg (N = 109)	Letrozole 5 mg (N = 102)
Maternal age at oocyte retrieval (y), mean (SD)	33.3 (3.96)	33.7 (3.67)	32.9 (4.22)
BMI (kg/m^2^), mean (SD)	21.8 (3.10)	21.3 (2.63)	21.5 (2.59)
Ethnicity, n (%)			
Han	207 (98.1%)	105 (96.3%)	101 (99.0%)
Other	4 (1.90%)	3 (2.75%)	0 (0.00%)
Residency, n (%)			
No	208 (98.6%)	107 (98.2%)	97 (95.1%)
Yes	3 (1.42%)	2 (1.83%)	5 (4.90%)
Gravidity, n (%)			
0	113 (53.6%)	59 (54.1%)	53 (52.0%)
1	56 (26.5%)	23 (21.1%)	23 (22.5%)
≥2	42 (19.9%)	27 (24.8%)	26 (25.5%)
Primiparous, n (%)	182 (86.3%)	99 (90.8%)	90 (88.2%)
Infertility cause, n (%)			
Tubal	95 (45.0%)	56 (51.4%)	58 (56.9%)
Ovulatory	14 (6.64%)	6 (5.50%)	5 (4.90%)
Uterine	10 (4.74%)	4 (3.67%)	3 (2.94%)
Male	52 (24.6%)	33 (30.3%)	29 (28.4%)
Unexplained	17 (8.06%)	5 (4.59%)	4 (3.92%)
Duration of infertility (y), median (IQR)	3 (2 - 5)	4 (2 - 6)	4 (2 - 6)
Antral follicle count, median (IQR)	8 (5 - 11)	8 (5 - 10.5)	7 **(3 - 10)**^b^

b Significantly (P < 0.05) different from GnRH antagonist group.

BMI, body mass index; GnRH, gonadotropin-releasing hormone; IQR, interquartile range; SD, standard deviation.

Bold values indicate statistical significance.

### Hormone profiles

**Figure 2:**
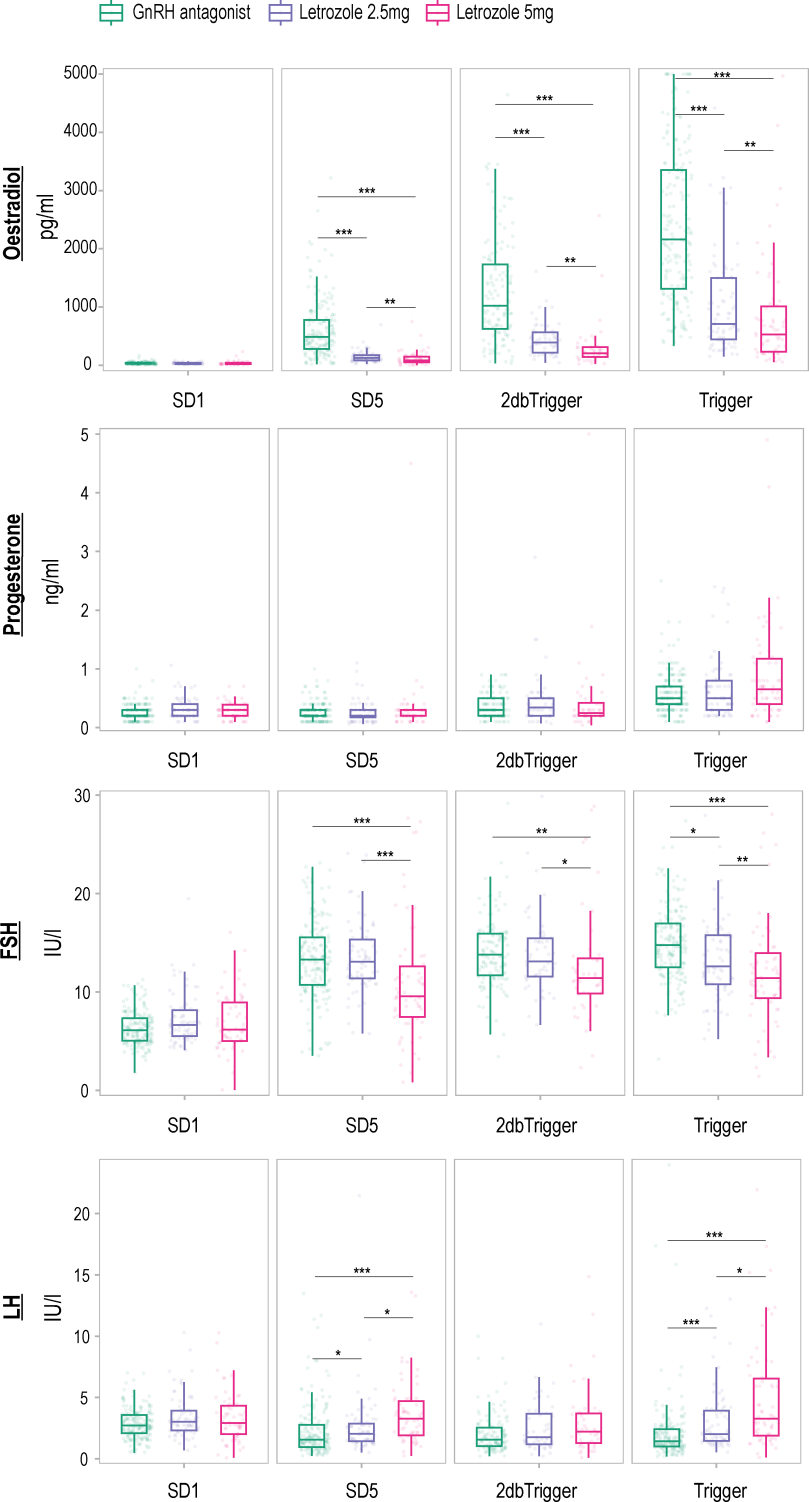
Hormone profiles measured in an antagonist protocol with and without letrozole co-treatment. Levels are shown on stimulation day 1 (SD1), 5-6 days after the stimulation (SD5), 2-3 days before ovulation triggering (2dbTrigger), and on the day of the ovulation trigger (Trigger). The data are presented in a box plot, which visually displays the median, interquartile range (representing the middle 50% of the values), and the range (excluding any outliers). Time point differences are compared using a two-way mixed ANOVA on log-transformed concentrations, with a Bonferroni correction applied for multiple testing. Significant differences between time points are denoted as ‘*’ for P < 0.05, ‘**’ for P < 0.01, and ‘***’ for P < 0.001. *FSH* follicle-stimulating hormone, *GnRH* gonadotropin-releasing hormone, *LH* luteinizing hormone.

Hormone profiles were analyzed, and the results are depicted in **Figure 2** and **Table 2**. Baseline levels of oestradiol, progesterone, FSH, and LH did not differ significantly among the three groups (P > 0.05). However, letrozole treatment led to a significant reduction in oestradiol concentrations from stimulation day 5 onwards, with a more pronounced suppressive effect observed at a dose of 5 mg letrozole compared to 2.5 mg letrozole (P < 0.05). The AUC analysis also revealed a significant decrease in oestradiol levels during the follicular phase in the two letrozole groups, but there was no difference between the 2.5 mg and 5 mg letrozole groups (P > 0.05). Progesterone levels remained unaffected by letrozole treatment throughout the cycle (P > 0.05).

**Table 2:** Assessment of AUC for follicular phase endocrine parameters.

	GnRH antagonist Median (IQR)	Letrozole 2.5mg Median (IQR)	Letrozole 5mg Median (IQR)
Oestradiol (pg/ml × days)	6470 (3836 – 10219)	**1831 (1074 – 3274)**^a^	**1366 (799 – 2846)**^b^
Progesterone (ng/ml × days)	2.65 (1.88 – 3.58)	2.45 (1.68 – 3.68)	3.00 (2.00 – 5.15)
FSH (IU/l × days)	99.2 (85.2 – 119)	90.4 (75.9 – 113)	**81.5 (56.9 – 113)**^b^
LH (IU/l × days)	18.4 (13.5 – 23.7)	19.2 (15.0 – 26.0)	**28.9 (19.4 – 41.6)^b,c^**

Differences between the groups are assessed by conducting ANOVA on log-transformed AUC values with a Bonferroni correction for multiple comparisons.

a Significantly (P < 0.05) different from GnRH antagonist group.

b Significantly (P < 0.05) different from GnRH antagonist group.

c Significantly (P < 0.05) different from letrozole 2.5mg group.

AUC area under the curve, FSH follicle-stimulating hormone, GnRH gonadotropin-releasing hormone, IQR interquartile range, LH luteinizing hormone.

Bold values indicate statistical significance.

Furthermore, letrozole treatment resulted in a significant increase in LH concentrations on SD5 and trigger day, with the 5 mg letrozole group showing a more pronounced effect compared to the 2.5 mg group (P < 0.05). The AUC analysis revealed a significant difference in LH concentrations between the 5 mg letrozole group and the other two groups, with higher concentrations observed in the 5 mg letrozole group (P < 0.05).

Interestingly, we found a significantly lower FSH concentration in the 5 mg letrozole co-treatment group compared to the other two groups from stimulation day 5 onwards (P < 0.05). Additionally, the 5 mg letrozole co-treatment group showed lower FSH concentration throughout the follicular phase analyzed as AUC compared to the control group (P < 0.05). The 2.5 mg letrozole co-treatment group also had lower FSH concentrations compared to the control group on the trigger day (P < 0.05).

### Clinical outcomes

The ovarian stimulation outcomes are summarized in **Table 3**. During controlled ovarian stimulation, patients co-treated with letrozole received a lower dose of gonadotropins and had a shorter stimulation duration with a lower number of retrieved oocytes compared with the control group (P < 0.05). Notably, the administration of 5 mg letrozole resulted in a reduction in the gonadotropin dose compared to 2.5 mg (P < 0.05), without impairing the number of oocytes retrieved and subsequent embryo parameters (P > 0.05).

**Table 3:** Ovarian stimulation outcomes in an antagonist protocol with and without letrozole co-treatment.

	GnRH antagonist (N = 211)	Letrozole 2.5 mg (N = 109)	Letrozole 5 mg (N = 102)
Gonadotropin consumption (IU), mean (SD)	2029 (607)	**1417 (695)**^a^	**1117 (868)^b,c^**
Days of stimulation, mean (SD)	8.92 (1.92)	**7.87 (2.04)**^a^	**8.01 (3.38)**^b^
GnRH antagonist consumption (mg), mean (SD)	0.23 (0.48)	0.21 (0.32)	0.15 (0.25)
Endometrial thickness at hCG day (mm), mean (SD)	10.8 (2.68)	**10.1 (2.15)**^a^	**9.76 (2.88)**^b^
Follicles on hCG day (10-12 mm), mean (SD)	1.79 (1.78)	**0.96 (1.47)**^a^	**0.69 (1.27)**^b^
Follicles on hCG day (12-14 mm), mean (SD)	2.11 (2.27)	**1.29 (1.68)**^a^	**1.13 (1.84)**^b^
Follicles on hCG day (14-16 mm), mean (SD)	1.99 (2.23)	**1.56 (2.19)**^a^	**1.51 (2.67)**^b^
Follicles on hCG day (>16 mm), mean (SD)	4.28 (2.92)	**3.41 (2.27)**^a^	**3.35 (2.75)**^b^
Fertilization method, n (%)			
IVF	126 (59.7%)	**62 (56.9%)**^a^	**57 (55.9%)**^b^
ICSI	84 (39.8%)	**35 (32.1%)**	**41 (40.2%)**
IVF+ICSI	0 (0.00%)	**10 (9.17%)**	**4 (3.92%)**
Retrieved oocytes, mean (SD)	8.07 (5.22)	**6.41 (4.18)**^a^	**6.04 (4.94)**^b^
Mature oocytes, mean (SD)	6.60 (4.29)	**5.06 (3.37)**^a^	**4.86 (3.86)**^b^
Fertilized oocytes, mean (SD)	5.85 (3.98)	**4.53 (3.24)**^a^	**4.40 (3.52)**^b^
Cleaved embryos, mean (SD)	5.35 (3.67)	**4.24 (2.85)**^a^	**4.15 (3.38)**^b^
Viable embryos, mean (SD)	3.36 (2.23)	3.12 (1.98)	2.98 (2.51)
Blastocysts, mean (SD)	0.72 (1.30)	**0.27 (0.63)**^a^	**0.25 (0.68)**^b^
High quality embryos, mean (SD)	2.62 (2.35)	2.64 (2.08)	2.58 (2.48)
All cryopreserved embryos, mean (SD)	2.18 (2.40)	1.78 (1.93)	2.10 (2.40)

a Significantly (P < 0.05) different from GnRH antagonist group.

b Significantly (P < 0.05) different from GnRH antagonist group.

c Significantly (P < 0.05) different from letrozole 2.5mg group.

GnRH gonadotropin-releasing hormone, hCG human chorionic gonadotropin, ICSI intracytoplasmic sperm injection, IVF in vitro fertilization, SD standard deviation.

a Significantly (P < 0.05) different from GnRH antagonist group.

Bold values indicate statistical significance.

Pregnancy outcomes are summarized in **Table 4**. A total of 289 fresh ET cycles were performed, involving 486 embryos. Women in the letrozole 5 mg co-treatment group tended to have a higher number of embryos transferred in fresh ETs compared to the control group (P < 0.05). The live birth rates per fresh ET were 14.9% in the letrozole 5 mg group, 15.3% in the letrozole 2.5 mg group, and 24.2% in the control group, but the difference was not statistically significant (P > 0.05). A total of 278 FET cycles were performed, involving 420 embryos. The live birth rates per FET were comparable across the three groups (25.6% in the letrozole 5 mg group, 27.0% in the letrozole 2.5 mg group, and 26.4% in the control group; P > 0.05). Furthermore, the analysis of cumulative live birth rates showed 29.4% in the letrozole 5 mg group, 27.5% in the letrozole 2.5 mg group, and 33.6% in the control group, with no statistically significant difference observed (P > 0.05).

**Table 4:** Pregnancy outcomes in an antagonist protocol with and without letrozole co-treatment.

	GnRH antagonist (N = 211)	Letrozole 2.5 mg (N = 109)	Letrozole 5 mg (N = 102)
Fresh cycle outcomes			
Cycles	n = 157	n = 85	n = 47
Average number of embryos transferred, mean (SD)	1.59 (0.53)	1.73 (0.54)	**1.91 (0.58)**^b^
Endometrial thickness (mm), mean (SD)	12.1 (2.70)	12.1 (2.95)	11.7 (3.00)
Pregnancy rate per ET, n (%)	56 (35.7%)	20 (23.5%)	12 (25.5%)
Live birth rate per ET, n (%)	38 (24.2%)	13 (15.3%)	7 (14.9%)
Frozen-thawed cycle outcomes			
Cycles	n = 125	n = 63	n = 90
Average number of embryos transferred, mean (SD)	1.53 (0.47)	1.52 (0.53)	1.48 (0.51)
Pregnancy rate per ET, n (%)	54 (43.2%)	23 (36.5%)	25 (27.8%)
Live birth rate per ET, n (%)	33 (26.4%)	17 (27.0%)	23 (25.6%)
Cumulative outcomes			
Cumulative live birth rate, n (%)	71 (33.6%)	30 (27.5%)	30 (29.4%)
Pregnancy complications			
Hypertensive disorders in pregnancy, n (%)	2 (2.82%)	0 (0.00%)	0 (0.00%)
Gestational diabetes, n (%)	4 (5.63%)	0 (0.00%)	0 (0.00%)
Intrahepatic cholestasis of pregnancy, n (%)	0 (0.00%)	0 (0.00%)	0 (0.00%)
Placenta previa, n (%)	0 (0.00%)	0 (0.00%)	0 (0.00%)
Placental abruption, n (%)	0 (0.00%)	0 (0.00%)	0 (0.00%)
Preterm premature rupture of the membranes, n (%)	0 (0.00%)	0 (0.00%)	0 (0.00%)
**Singletons**	n = 65	n = 27	n = 26
Male/Female	113/100	100/100	82/100
Gestational age (weeks), mean (SD)	38.7 (1.64)	38.3 (0.97)	38.3 (1.24)
Birth weight (g), mean (SD)	3255 (423)	3277 (475)	3305 (488)
**Twins**	n = 12	n = 6	n = 8
Male/Female	200/100	100/100	300/100
Gestational age (weeks), mean (SD)	35.6 (2.07)	36.0 (2.00)	36.5 (0.71)
Birth weight (g), mean (SD)	2167 (425)	2350 (212)	3040 (226)

b Significantly (P < 0.05) different from GnRH antagonist group.

GnRH gonadotropin-releasing hormone, ET embryo transfer, SD standard deviation.

Bold values indicate statistical significance.

Regarding pregnancy complications, two cases of gestational hypertension and four cases of gestational diabetes were reported in the GnRH antagonist control group. However, no pregnancy complications were reported in the two letrozole groups. Additionally, there were no significant differences among the three groups in terms of gestational age and birth weight for both singleton and twin births (P > 0.05).

## Discussion

Through a single-center retrospective analysis, we discovered that letrozole 5 mg exhibited a more pronounced effect in suppressing oestradiol levels and upregulating LH levels during the follicular phase, while also reducing overall gonadotropin consumption. However, our investigation did not reveal any statistically significant difference in clinical outcomes when comparing letrozole 2.5 mg versus 5 mg daily. Consequently, both dosages appeared to yield comparable results in this patient population.

A significant decrease in oestradiol levels at trigger day has been observed in most studies using either 2.5 mg or 5 mg letrozole per day (9–14), as well as in studies employing higher dosages (10 mg or 20 mg) (15, 16). However, some studies, particularly in poor responders, have failed to find a significant association between letrozole and reduced oestradiol levels (17–21). The results from two recent randomized, double-blinded, placebo-controlled trials (RCTs) for normal responders have indicated a significant decline in oestradiol levels during both the follicular and luteal phases (6, 22). A prior investigation has explored the dose-dependent reduction of serum oestradiol levels through the administration of letrozole, commencing on the day of oocyte retrieval in patients with high-risk OHSS (23). Our study adds to this body of literature by demonstrating that letrozole significantly suppressed oestradiol levels during the follicular phase in women with normal ovarian response, with 5 mg letrozole exhibiting a more pronounced effect than 2.5 mg. Furthermore, letrozole also exhibited an upregulating effect on LH levels during the follicular phase, consistent with the findings of the aforementioned trials (6, 22). Interestingly, our findings revealed a significantly lower FSH concentration in the letrozole co-treatment groups, contradicting the results of other studies that have reported increased FSH levels after letrozole administration (6, 22). The letrozole-induced promotion of follicular development is likely mediated by a dual mechanism involving elevated endogenous FSH levels and augmented follicular sensitivity to exogenous FSH (24). Our investigation has underscored the importance of the latter mechanism.

A recent meta-analysis, including eight studies with a total of 768 participants, concluded that the administration of letrozole did not significantly affect the consumption of gonadotropins in normal responders. However, there was a significant difference in favor of letrozole in the number of oocytes retrieved, based on data from eight studies with a total of 804 participants (8). It is important to note that the studies included in this analysis have suffered from small sample sizes and heterogeneity in the selected protocols, gonadotropin dosages, and trigger criteria. Our study demonstrated that patients co-treated with letrozole required lower doses of exogenous gonadotropins and shorter stimulation durations. Although we did not find significant differences in the number of usable embryos, we did observe a potential reduction in the number of retrieved oocytes with the use of letrozole. However, it was noteworthy that women co-treated with letrozole 5 mg required lower exogenous gonadotropin consumption without compromising the number of retrieved oocytes and subsequent embryo parameters compared to those treated with letrozole 2.5 mg.

The current literature on the impact of letrozole on live birth outcomes among normal responders remains limited. A recent RCT comprising 129 participants revealed equivalent ongoing pregnancy rates following the administration of letrozole (6), while a retrospective study involving 252 individuals found no discernible effect of letrozole on the cumulative live birth rate among normal responders (25). Our results showed that there were no significant differences in live birth outcomes between the groups administered with letrozole and the control group. Moreover, there were no discernible differences observed in the live birth outcomes between the two doses of letrozole (2.5mg and 5mg).

Importantly, no pregnancy or neonatal complications were reported in either letrozole group. Previous data have indicated that letrozole during IVF does not pose an elevated risk of major congenital anomalies or compromise neonatal outcomes compared to natural cycles, supporting the safety profile of letrozole (26, 27). Furthermore, supraphysiological oestradiol concentrations during ovarian stimulation are known to have deleterious effects on various physiological processes, including leptin regulation, the coagulation system, and early placental development (7), contributing to adverse obstetric outcomes. Letrozole treatment may provide an optimal uterine environment, potentially leading to better placentation and improved maternal outcomes. However, further studies with larger sample sizes are needed to confirm these observations.

This analysis was subject to certain limitations. Firstly, the retrospective design of the study introduced the possibility of selection bias regarding the choice of IVF protocol. Secondly, the limited sample size of live births might have only captured strong associations. Thirdly, the dosages of gonadotropins used in this study were not strictly controlled. Lastly, hormone profiles in the luteal phase were not assessed through blood tests. Nevertheless, this study fills a gap in the literature by comparing the clinical outcomes and endocrinological characteristics of different doses of letrozole (2.5 mg or 5 mg daily) in IVF patients.

## Conclusion

Our study contributes to the current understanding of letrozole use in an antagonist protocol for IVF/ICSI, indicating that co-treatment with either 2.5 mg or 5 mg of letrozole yields similar pregnancy outcomes. A stimulation protocol with a minimum of 2.5 mg letrozole per day for at least 5 days appears appropriate to ensure significant suppression of oestradiol in most women, while 5 mg letrozole demonstrates more efficiency in altering endocrinological characteristics and reducing total gonadotropin consumption.

These findings provide valuable insights into the efficacy of letrozole in IVF treatment, guiding clinicians in optimizing treatment strategies for patients undergoing assisted reproductive technology treatments. However, larger high-quality studies are needed to explore the appropriate dose of letrozole co-treatment without any detrimental effects on clinical outcomes.

## Data availability statement

The original contributions presented in the study are included in the article. Further inquiries can be directed to the corresponding authors.

## Ethics statement

The studies involving humans were approved by the Institutional Review Board of Shanghai Ninth People’s Hospital of Shanghai Jiao Tong University School of Medicine (Shanghai, China). The studies were conducted in accordance with the local legislation and institutional requirements. The ethics committee/institutional review board waived the requirement of written informed consent for participation from the participants or the participants’ legal guardians/next of kin because the data were deidentified and the analyses were retrospective in nature.

## Author contributions

JL: Conceptualization, Formal Analysis, Writing – original draft, Writing – review & editing. FW: Data curation, Writing – review & editing. KZ: Formal Analysis, Writing – original draft. YZ: Data curation, Writing – review & editing. BW: Writing – review & editing. QZ: Funding acquisition, Supervision, Writing – review & editing. JYL: Conceptualization, Funding acquisition, Supervision, Writing – review & editing.
